# Effect of the haemoglobin level on neurologic outcome in patients with severe traumatic brain injury

**DOI:** 10.1186/cc14415

**Published:** 2015-03-16

**Authors:** K Balvers, MR Wirtz, C Rourke, S Eaglestone, K Brohi, S Stanworth, C Gaarder, JC Goslings, NP Juffermans

**Affiliations:** 1 Academic Medical Center, Amsterdam, the Netherlands; 2 Blizard Institute, Queen Mary University of London, UK; 3 John Radcliffe Hospital, Oxford, UK; 4 Oslo University Hospital, Oslo, Norway

## Introduction

Anaemia in patients with severe traumatic brain injury (TBI) may worsen neurologic outcome. The aim of this study was to determine the association of haemoglobin level (Hb) with neurologic outcome and to determine a transfusion threshold which may be used in future transfusion trials aimed at improving neurologic outcome in TBI patients.

## Methods

A substudy of the prospective multicentre Activation of Coagulation and Inflammation in Trauma (ACIT) II study was performed on subjects recruited between January 2008 and December 2014. All adult trauma patients admitted to a level 1 trauma centre with severe traumatic brain injury (AIS head ≥3), ICU admission and available Hb levels on admission were selected for analysis. The primary outcome was the cognitive functioning of patients as determined by an estimated Glasgow Outcome Scale (GOS) on discharge. Anaemia was defined as a Hb level of ≤9 g/dl (severe anaemia) or ≤10 g/dl (moderate anaemia) within the first 24 hours post injury. Multivariate logistic regression models were used to determine the association between anaemia and neurologic outcome. The receiver operating characteristic curve and the Youden Index were used to determine an optimal transfusion threshold.

## Results

Of a total of 261 TBI patients, 61 patients (23%) fell below the threshold for severe anaemia and 101 patients (39%) had moderate anaemia within the first 24 hours. In a model adjusted for all relevant confounders, a Hb level of ≤9 g/dl was associated with a poor neurologic outcome (OR = 2.572, 95% CI = 1.058 to 6.250), whereas an Hb level of ≤10 g/dl was not. A Hb level of ≤10.3 g/dl had a sensitivity of 82% and a specificity of 62% to predict poor neurologic outcome in TBI patients.

## Conclusion

In TBI patients a Hb level of ≤9 g/dl is associated with poor neurologic outcome. A transfusion threshold of ≤10.3 g/dl may be a reasonable target to be tested in future transfusion trials aimed at improving neurologic outcome of TBI patients.

## Acknowledgements

All of the author institutes are affiliated members of the International Trauma Research Network (INTRN) and as such this work represents a combined output resulting from this Network.

